# Serum eosinophil-derived neurotoxin: a new promising biomarker for cow’s milk allergy diagnosis

**DOI:** 10.1038/s41390-024-03260-x

**Published:** 2024-05-27

**Authors:** Wael A. Bahbah, Ahmed S. Abo Hola, Hanan M. Bedair, Eman T. Taha, Heba M. S. El Zefzaf

**Affiliations:** 1 Department of Pediatrics, Faculty of Medicine, Menoufia University, Shebin El-Kom, Egypt; 2 Department of Clinical Pathology, National Liver Institute, Menoufia University, Shebin El-Kom, Egypt; 3 Ministry of Health, Shebin El-Kom, Menoufia Egypt

## Abstract

**Background:**

Cow’s Milk Allergy (CMA) diagnosis is often a challenge due to the non-specific nature of symptoms and lack of a confirmatory diagnostic test. To our knowledge no previous studies investigated serum Eosinophil-Derived Neurotoxin (sEDN) in CMA. So, we aimed to assess the role of sEDN in CMA diagnosis.

**Methods:**

Forty-five infants with CMA were compared to 45 infants with functional gastrointestinal disorders (FGIDs) and 45 healthy controls. For all participants, Cow’s Milk-related Symptom Score (CoMiSS) was documented, and sEDN level with hematological parameters were measured before starting elimination diet.

**Results:**

Receiver operation characteristic (ROC) curve identified sEDN > 14 ng/mL and CoMiSS > 9 as the optimal cut-off points to discriminate CMA from other groups with sensitivity 86.67%, 97.78% and specificity 60.00%, 78.89% respectively. Additionally, absolute neutrophil count (ANC) showed the highest sensitivity and specificity (80.0% and 78.89%) among hematological parameters. Although CoMiSS and ANC showed a significant positive correlation with sEDN in CMA group, CoMiSS was the only significant predictor for sEDN in multivariate linear regression.

**Conclusions:**

sEDN showed high sensitivity in discriminating infants with and without CMA. Therefore, it is suggested as a potential biomarker for CMA diagnosis. Also, ANC should be closely monitored in these infants.

**Impact:**

- CMA presents with high heterogeneity, which complicates the diagnosis especially non-IgE-mediated and mixed types. So, oral food challenge continues to be the gold standard for its diagnosis.
- ROC curve identified CoMiSS > 9 as the best cut-off point to identify CMA. However, CoMiSS is a good awareness tool for CMA but not a diagnostic tool.
- sEDN level was significantly higher in infants with CMA with a good diagnostic performance in differentiating them than those without CMA. So, it is suggested as a potential biomarker for CMA diagnosis.
- ANC could have a role in CMA diagnosis and differentiating it from FGIDs.

## Introduction

Cow’s milk allergy (CMA) is the most prevalent cause of food allergy (FA) in infants and children younger than 3 years.^1^ Less than 5.0% is the reported prevalence of CMA, and according to the EuroPrevall data, the prevalence is as low as 0.54%.^2^ Despite the globally reported rise in CMA prevalence, the pathogenesis remains unclear. The pathophysiology of CMA may involve T regulatory cells, antigen-specific T cells, and certain mediators released by T and B lymphocytes.^3^

Symptoms occurring up to two hours after cow’s milk (CM) consumption could suggest an IgE-mediated allergic reaction.^4^ On the other hand, persistent symptoms that mainly affect the gastrointestinal (GI) tract and occur 2 to 4 weeks after CM consumption could be indicative of a non-IgE-mediated allergy. Non-IgE-mediated CMA not only cause proctocolitis, enteropathy, food protein-induced enterocolitis syndrome (FPIES), but also can cause nonspecific GI manifestations such as abdominal pain, dyspepsia, regurgitation, or persistent constipation with varied severity of these reported symptoms.^5^

It can be difficult to distinguish non-IgE-mediated CMA from functional gastrointestinal disorders (FGIDs), gastro-esophageal reflux disease (GERD), or other problems such as neurological, metabolic, endocrine, anatomic, and infectious diseases, since many infants with these conditions exhibit a combination of symptoms, in addition to lack of optimal diagnostic tests and lack of biomarkers.^1,6,7^

A prompt diagnosis of CMA is crucial to improve faltering growth and quality of life.^8^ However, over-diagnosing CMA and improper linking of the above-mentioned conditions with CMA results in unnecessary dietary eliminations, burdens caregivers, raises health care costs, and may lead to nutritional deficiencies.^9^

As a clinical tool, the Cow’s Milk-related Symptom Score (CoMiSS) was developed to raise awareness that symptoms could be related to CM. Although a cut-off score ≥10 could suggest CMA, CoMiSS is not a reliable diagnostic tool for CMA on its own.^10^

As there are no disease-specific markers, complete blood count (CBC) parameters which are easily measured in routine tests and include total leukocyte count (TLC), platelet count, mean platelet volume (MPV), eosinophil percentage, and neutrophil-lymphocyte ratio (NLR), may be considered as potential diagnostic biomarkers for inflammation. Recent research investigated MPV and NLR as inflammatory markers in a variety of diseases, such as ulcerative colitis, acute pancreatitis, and myocardial infarction.^11,12^

Eosinophils have proven to be the best marker of allergic inflammation and are considered the major effector cells of allergic process. Many granule proteins, including Eosinophil-Derived Neurotoxin (EDN), are released extracellularly when eosinophils are activated.^13^ EDN, an RNase with wide antiviral and antibacterial action, is released from active eosinophils during an inflammatory process. It is selectively released from cytotoxic proteins, and this release could promote more leukocyte activation. EDN has already been used in many studies before to monitor and control allergic rhinitis and asthma.^14^

To the best of our knowledge no previous studies investigated serum EDN (sEDN) in CMA. Therefore, the aim of our study was to investigate the sEDN levels, absolute neutrophil count (ANC), absolute lymphocyte count (ALC), MPV, and NLR, in addition to CoMiSS in infants with CMA compared to those of FGIDs and healthy controls, and to determine the suitability of sEDN and hematological parameters as biomarkers in CMA diagnosis. Also, to evaluate the relationship of sEDN with hematological parameters and the available clinical score (CoMiSS). To date, these parameters have not been simultaneously evaluated in infants with CMA.

## Methods

This cross-sectional study was conducted on pediatric gastroenterology and clinical nutrition clinic in Menoufia University Hospital from November 2022 to September 2023. A total of 144 infants with symptoms suggestive of CMA or FGIDs (the most frequently presenting symptoms were diarrhea, regurgitations, colic, bloody diarrhea, and constipation), in addition to apparently healthy controls were enrolled in the study. In accordance with the Helsinki Declaration of Principles, the Institutional Review Boards (IRB) of the Menoufia Faculty of Medicine approved the present study (ID number: 11/2022 PEDI 23). Our participants completed the study after a written consent from their parents/guardians.

### Study population

54 infants who presented with symptoms suspecting CMA before starting cow milk free diet (CMFD) were initially included as CMA group (Group 1), then CMFD was introduced for 4 weeks to all of them (breastfeeding was continued while mothers instructed to avoid ingestion of all CM products in breast-fed infants, and Amino Acid-based Formula (AAF) was prescribed in formula-fed infants), followed by oral food challenge (OFC) according to European Society for Pediatric Gastroenterology Hepatology and Nutrition (ESPGHAN) Guideline for Diagnosis and Management of CMA.^5^ Only those with a positive OFC (defined by improvement of symptoms following 4 weeks of CMFD with symptoms recurrence upon reintroduction of CM) fulfilled the diagnosis of CMA and completed the study as CMA group (*n* = 45).

**Group 2** included 45 age and sex matched infants who fulfilled the diagnosis of FGIDs according to Rome IV criteria last update (2016) of infantile FGIDs,^15^ in addition to 45 apparently healthy infants (like distribution of age and sex) as the control group (Group 3). So, a total of 135 infants were enrolled in this study in 3 groups.

Our inclusion criteria for all recruited infants included: age 0–12 months with no nutritional or pharmacological interventions, and without having any acute illness or hospital admission in the month prior to enrollment. Infants were excluded if they had history of anaphylaxis following CM ingestion, were receiving partially hydrolyzed Formula, extensively hydrolyzed Formula (ehF), or AAF on initial presentation, their parents refused OFC, had GI malformations, or had any chronic diseases.

### Clinical assessment

For all infants, full history including age, mode of delivery, consanguinity, family history of CMA, type of feeding (breastfeeding, formula feeding or mixed feeding), presenting symptoms, and systems affected (GI only, GI, and respiratory, GI and skin or GI, respiratory, and skin) were documented. Also, CoMiSS was completed on the initial visit according to the updated CoMiSS (2022)^10^ (Table 1). Anthropometric measures were taken including weight, length, and weight for length according to Z score growth references for Egyptian children from birth up to 5 years.^16^

**Table 1:** The updated CoMiSS (2022).

Symptom				Score
Crying ^1^ assessed by parents & without any obvious cause ≥1 week	≤1 h/day			0
	1–1.5 h/day			1
	1.5–2 h/day			2
	2 to 3 h/day			3
	3 to 4 h/day			4
	4 to 5 h/day			5
	≥5 h/day			6
Regurgitation ^1^ ≥ 1 week	0–2 episodes/day			0
	≥ 3– ≤ 5 of volume < 5 mL			1
	> 5 episodes of >5 mL			2
	> 5 episodes of ± half of the feed in < half of the feeds			3
	continuous regurgitations of small volumes > 30 min after each feed			4
	regurgitation of half to complete volume of a feed in at least half of the feeds			5
	regurgitation of the complete feed after each feeding			6
Stools ^1^ Brussels Infant and Toddlers Stool Scale (BITSS) No change ≥ 1 week	hard stools			4
	formed stools			0
	loose stools			4
	watery stools			6
Skin symptoms	Atopic eczema ≥ 1 week			0 to 6
		Head neck trunk	Arms hands legs feet	
	Absent	0	0	
	Mild	1	1	
	Moderate	2	2	
	Severe	3	3	
	Acute urticaria ^1^ and/or angioedema ^1^ (no 0/yes 6)			0 or 6
Respiratory symptoms ^1^ ≥1 week	no respiratory symptoms			0
	slight symptoms			1
	mild symptoms			2
	severe symptoms			3
Additional information to consider				
Worsening of existing eczema might be indicative of CMA				
Worsening of existing eczema might be indicative of CMA				

^1^In the absence of infectious disease. If urticaria/angioedema can be directly related to cow’s milk (e.g., drinking milk in the absence of other food) this is strongly suggestive of CMA.

### Laboratory assessment

By sterile venipuncture, 5 mL blood sample were taken and then divided into two tubes; three mL plain tube with clot activator for serum preparation of sEDN and CM specific IgE levels measurement that was collected and stored at −80 °C until assayed. The determination of serum specific IgE were carried out using IMMULITE 2000 which is automated immunological analyzer (Siemens Healthcare GmbH, Erlangen, Germany),by chemiluminescent competitive immunoassay method (CM specific IgE level ≥ 0.35 kU/L was considered positive). sEDN was measured by Enzyme linked Immunosorbent Assay using ELISA Kit with catalog number E4029hu supplied by Bioassay Technology Laboratory (BT LAB, Zhejiang, China).

Another two mL were withdrawn in K2 EDTA for CBC. The measurement of CBC with differential was done using Sysmex XN-1000, automated Hematology Analyzer which utilizes the fluorescent flowcytometry and hydrodynamic focusing technologies (Sysmex, Kobe, Japan). All parameters of CBC are recorded including ANC, ALC and MPV, then we calculate NLR by dividing neutrophilic count to lymphocytic count.

### Statistical analysis

Data was fed to the computer and analyzed using IBM SPSS software package version 20.0. (Armonk, NY: IBM Corp). The Chi-square test was applied to compare between two groups. Alternatively, Fisher Exact correction test was applied for binomial data when more than 20% of the cells have expected count less than 5, and Monte Carlo correction test was applied when more than 20% of the cells have expected count less than 5. Quantitative data were expressed as minimum and maximum, mean ± standard deviation for data with a normal distribution, or median and inter quartile range (IQR). One way ANOVA test was used for comparing the three studied groups and followed by Post Hoc test (Tukey) for pairwise comparison while Kruskal Wallis test was used to compare different groups for not normally distributed quantitative variables and followed by Post Hoc test (Dunn’s for multiple comparisons test) for pairwise comparison. The spearman’s coefficient was used to determine the correlation between sEDN, CoMiSS and hematological parameters. Linear regression analysis was done to detect the most independent factor affecting sEDN result. The receiver operating characteristic (ROC) curve was generated to denote the diagnostic performance of the test to discriminate between infants with and without CMA. *P*-value ≤ 0.05 was considered Significant.

Based on review of past literature,^17^ the least sample size calculated using statistics and sample size pro program version 6 was 40 subjects increased by 10% to avoid dropout with the power of study 80% and confidence level 95%. Therefore, the minimum sample size was 44 subjects.

## Results

The median age of studied infants was 8 months in CMA group versus 8 months in FGIDs group, and 9 months in the control group with no significant difference between the 3 groups. Infants in CMA group showed significantly lower median z score of weight and weight for length than the other 2 groups (*p* < 0.001) with no significant difference between FGIDs and control groups in all anthropometric measurements. In CMA group, 15 cases (33.3%) were underweighted, and 12 cases (26.7%) were wasted. Table 2 shows clinical characteristics of the three studied groups.

**Table 2:** Clinical characteristics among the three studied groups.

	Cow’s milk allergy group (*n* = 45)	Functional GI disorders group (*n* = 45)	Control group (*n* = 45)	*p*	Significance between groups		
					CMA vs FGIDs	CMA vs control	FGIDs vs control
**Age (months)**							
Median (IQR)	8.0(6.0–10.0)	8.0(6.0–11.0)	9.0(7.0–12.0)	0.174	> 0.05	> 0.05	> 0.05
**Sex**							
Male	26(57.8%)	32(71.1%)	23(51.1%)	0.143	> 0.05	> 0.05	> 0.05
Female	19(42.2%)	13(28.9%)	22(48.9%)				
**Positive Family history of CMA**	7(15.6%)	0(0.0%)	0(0.0%)	< 0.001^1^	0.012^1^	0.012^1^	-
**Type of feeding**							
Breastfeeding	8(17.8%)	13(28.9%)	11(24.4%)	0.046^1^	0.015^1^	0.036^1^	0.877
Formula feeding	33(73.3%)	20(44.4%)	22(48.9%)				
Mixed feeding	4(8.9%)	12(26.7%)	12(26.7%)				
**Weight for age z score**							
median (IQR)	−1.0(−2.0 to 0.0)	0.0(−1.0 to 0.10)	0.0(−1.10 to 0.10)	0.001^1^	0.001^1^	0.003^1^	0.672
Underweight	15(33.3%)	0(0%)	0(0%)	< 0.001^1^	< 0.001^1^	< 0.001^1^	–
Normal	30(66.7%)	45(100%)	45(100%)				
**Length for age z score**							
Median (IQR)	0.10(0.0–0.80)	0.0(0.0–0.20)	0.0(−1.0 to 0.70)	0.252	> 0.05	> 0.05	> 0.05
Normal	45(100%)	45(100%)	45(100%)	-	-	-	-
**Weight for length z score**							
Median (IQR)	−1.50(−3.0 to 0.0)	0.0(−1.40 to 0.80)	0.0(−1.0 to 1.0)	< 0.001^1^	< 0.001^1^	< 0.001^1^	0.680
Normal	33(73.3%)	45(100%)	45(100%)	< 0.001^1^	< 0.001^1^	< 0.001^1^	–
Wasted	12(26.7%)	0(0%)	0(0%)				

*CMA* Cow’s milk allergy, *FGIDs* Functional gastrointestinal disorders, *GI* Gastrointestinal.

Normal weight = weight for age between −2 and +1 standard deviation SD on Egyptian Z score.

Underweight = weight for age < −2 SD on Egyptian Z score.

Normal length = length for age between −2 and +3 SD on Egyptian Z score.

Normal weight for length = weight for length between −2 and +1 SD on Egyptian Z score.

Wasted = weight for length < −2 SD on Egyptian Z score.

*IQR* Inter quartile range.

^1^Statistically significant at *p* ≤ 0.05.

### Clinical presentation

Among infants in CMA group, GI system affection was the most prevalent either alone in 24 cases (53.34%) or associated with other systems either GI and respiratory, GI and skin or GI, respiratory, and skin in 22.22%, 13.33% and 11.11% respectively. Most infants presented with more than one symptom, diarrhea was the most frequently reported symptom in 75.55%, followed by regurgitation in 71.11%, bloody diarrhea in 31.11%, then constipation in 20%.

Infant regurgitation was the most prevalent in FGIDs group presented in 16 infants (35.56%), followed by infant colic in 11 infants (24.44%), then functional constipation in 8 infants (17.78%), and 10 infants (22.22%) presented with a combination of FGIDs.

### CoMiSS evaluation

Median total CoMiSS was significantly higher in CMA group than FGIDs group and control group (14, 9 and 4 respectively; *p* < 0.001). Also, total CoMiSS was significantly higher in the FGIDs group than the control group (*p* < 0.001). Regarding CoMiSS parameters, stool changes showed significant difference between CMA group compared to FGIDs and control groups, also between FGIDs and control groups. However, regurgitation and crying showed no significant difference between CMA group and FGIDs (*p* = 0.114 and 0.968 respectively) with a high statistical significance between each of them and the control group (p < 0.001). (Table 3)

**Table 3:** CoMiSS and laboratory parameters among the three studied groups.

	Cow’s milk allergy group (*n* = 45)	Functional GI disorders group (*n* = 45)	Control group (*n* = 45)	*p*	Significance between groups		
					CMA vs FGIDs	CMA vs control	FGIDs vs control
**CoMiSS**							
**Respiratory**							
Median (IQR)	1.0 (1.0–1.0)	0.0 (0.0–0.0)	0.0 (0.0–0.0)	< 0.001^1^	< 0.001^1^	< 0.001^1^	N/A
**Crying**							
Median (IQR)	3.0 (2.0–4.0)	3.0 (2.0–3.0)	1.0 (1.0–2.0)	< 0.001^1^	0.968	< 0.001^1^	< 0.001^1^
**Regurgitation**							
Median (IQR)	2.0 (2.0–3.0)	2.0 (2.0–3.0)	1.0 (1.0–2.0)	< 0.001^1^	0.114	< 0.001^1^	< 0.001^1^
**Stool**							
Median (IQR)	4.0 (4.0–6.0)	4.0 (2.0–4.0)	0.0 (0.0–2.0)	< 0.001^1^	< 0.001^1^	< 0.001^1^	< 0.001^1^
**Skin**							
Median (IQR)	2.0 (2.0–3.0)	0.0 (0.0–0.0)	0.0 (0.0–0.0)	< 0.001^1^	< 0.001^1^	< 0.001^1^	0.687
**Total score**							
Median (IQR)	14.0(12.0 – 14.0)	9.0(7.0–10.0)	4.0(4.0–5.0)	< 0.001^1^	< 0.001^1^	< 0.001^1^	< 0.001^1^
**Laboratory parameters**							
**Mean platelet volume (femtolitre)**							
Median (IQR)	9.20 (8.0–11.0)	9.0 (8.0 – 10.0)	8.0 (8.0–9.0)	0.024^1^	0.300	0.007^1^	0.096
**Total leukocyte count** (× 10^3^/mm)							
Median (IQR)	10.40(8.2–13.0)	9.0(7.3 – 12.0)	7.80(6.2 –9.7)	0.011^1^	0.309	0.003^1^	0.052
**Absolute lymphocyte count (**cells/ mm^3^)							
Median (IQR)	4500(3600–5800)	4000(3500–4400)	3800(3400–4500)	0.008^1^	0.021^1^	0.003^1^	0.511
**Absolute neutrophil count (**cells/ mm^3^)							
Median (IQR)	5900(4800–6900)	3700(3500–3900)	3700(3300–3900)	< 0.001^1^	< 0.001^1^	< 0.001^1^	0.881
**Neutrophil lymphocyte ratio**							
Median (IQR)	1.19 (1.0–1.44)	0.90 (0.84–1.11)	1.0 (0.76–1.19)	< 0.001^1^	0.001^1^	0.003^1^	0.611
**Absolute eosinophilic count**							
(cells/ mm^3^) Median (IQR)	600.0(400–1100)	600.0 (300–900)	600.0 (400–800)	0.270	> 0.05	> 0.05	> 0.05
**Serum Eosinophil-Derived Neurotoxin (ng/ml)**							
Median (IQR)	27.0(18.0–42.0)	13.0(9.0–28.0)	14.0(9.0–26.0)	< 0.001^1^	< 0.001^1^	< 0.001^1^	0.614

*CMA* Cow’s milk allergy, *FGIDs* Functional gastrointestinal disorders, *GI* Gastrointestinal, *CoMiSS* Cow milk related symptom score, *N/A* Not applicable, *IQR* Inter quartile range, *p* *p*-value for comparing between the studied groups.

^1^Statistically significant at *p* ≤ 0.05.

**Fig. 1:**
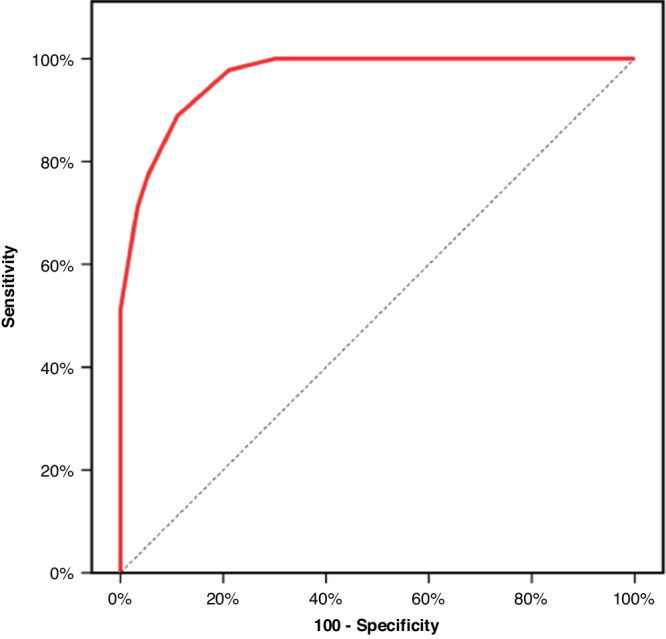
ROC curve for total CoMiSS. ROC curve identified the score of > 9 as the best cut-off point **to** discriminate CMA group (*n* = 45) vs FGIDs and Control groups (*n* = 90). Area under the curve (AUC): 0.964, with a sensitivity of 97.78%, specificity of 78.89%, PPV of 69.8, and NPV of 98.6.

The ROC curve analysis of CoMiSS identified score > 9 as the best cut-off for differentiating between infants with CMA and without CMA (FGIDs and control groups) with 97.78% sensitivity, 78.89% specificity, and area under the curve (AUC): 0.964. Figure 1 shows the ROC curve for total CoMiSS to discriminate CMA group (*n* = 45) versus FGIDs and Control groups (*n* = 90).

### CM specific IgE testing and hematological parameters

66.7% of CMA patients had non IgE mediated CMA and CM specific IgE testing was positive in 15 infants (33.3%) in CMA group. Regarding hematological parameters, median ALC, ANC and NLR showed highly statistically significant difference in CMA group than FGIDs and control groups (*p* = 0.008, *p* < 0.001 and *p* < 0.001 respectively). Although the values were within the normal range in all three groups, there was a statistically significant difference in the median total leukocyte count (TLC) and median MPV between the CMA and control groups, but no significant difference between CMA and FGIDs groups. Conversely, there was no statistically significant difference between the three groups with respect to absolute eosinophil count (*p* = 0.270). These laboratory parameters are shown in Table 3.

**Fig. 2:**
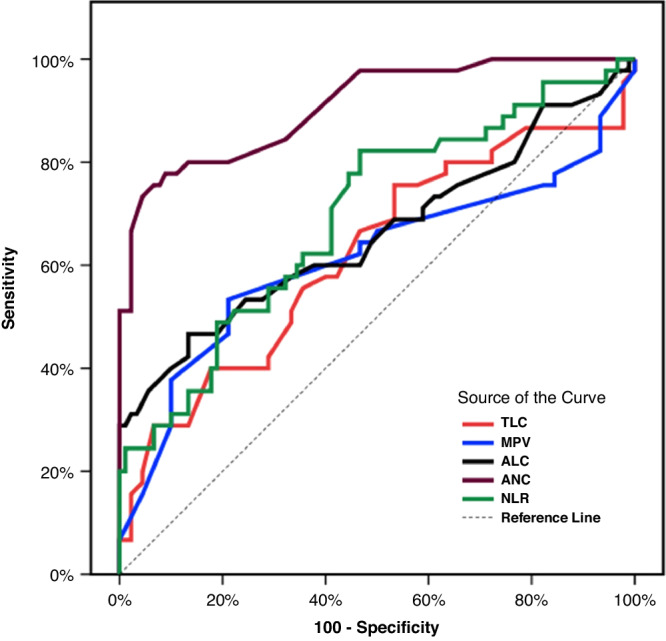
ROC curve for hematological parameters. ROC curve analysis of hematological parameters to discriminate CMA group (*n* = 45) vs FGIDs and Control groups (*n* = 90) revealed high sensitivity and specificity of ANC (sensitivity 80%, specificity 78.89%, and AUC: 0.909). In contrast, NLR, TLC, MPV and ALC showed low sensitivity and specificity (sensitivity 0.71.11%, 66.67%, 62.22% and 60%, specificity 58.89%, 53.33%, 53.33%, and 53.33% respectively).

The ROC curve analysis of hematological parameters revealed high sensitivity and specificity of ANC in infants with CMA than infants without CMA (sensitivity 80%, specificity 78.89%, and AUC: 0.909). In contrast, NLR, TLC, MPV and ALC showed low sensitivity and specificity (sensitivity 71.11%, 66.67%, 62.22% and 60%, specificity 58.89%, 53.33%, 53.33%, and 53.33% respectively). (Fig. 2)

### Serum Eosinophil-Derived Neurotoxin

**Fig. 3:**
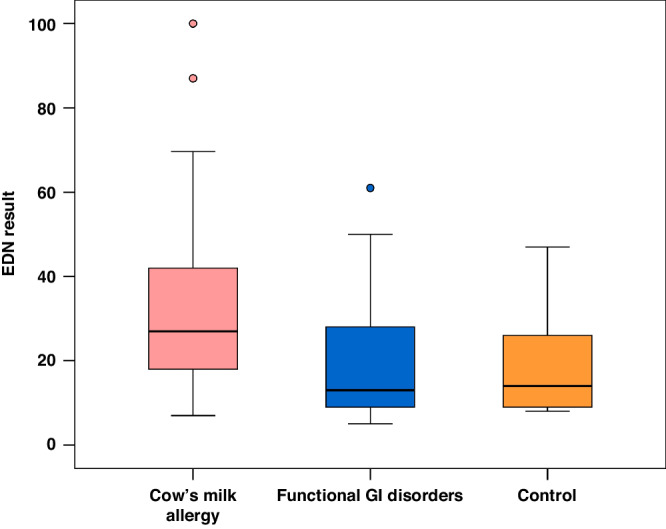
Serum Eosinophil-Derived Neurotoxin level among the 3 studied groups. Median (IQR) of sEDN (ng/mL) in the 3 studied groups.

**Fig. 4:**
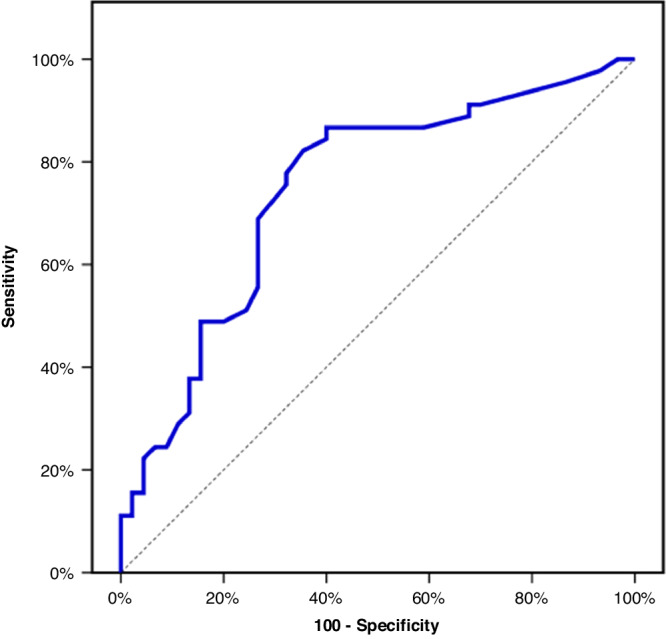
ROC curve for serum eosinophil-derived neurotoxin. ROC curve identified the score of > 14 ng/mL as the best cut-off point **to** discriminate CMA group (n = 45) vs FGIDs and Control groups (n = 90). Area under the curve (AUC): 0.754, with 86.67% sensitivity, 60.00% specificity, PPV of 52.0, and NPV of 98.6.

The median sEDN level in the CMA group was significantly higher than that in the FGIDs and control groups (27.0 versus 13.0 and 14.0 ng/mL, respectively; *P* < 0.001) with no significant difference between FGIDs and control groups (Table 3) (Fig. 3). Also, no statistically significant difference in the median sEDN level was found across CMA patients exhibiting positive and negative CM-specific IgE (31, 26 and *p* = 0.462 respectively). The ROC curve analysis revealed sEDN level > 14 ng/mL as the optimal cut-off level for differentiating between infants with and without CMA, (sensitivity 86.67%, specificity 60.00%, and AUC: 0.754) (Fig. 4).

Table 4 illustrates comparison between Validity of CoMiSS, sEDN, TLC, MPV, ALC, ANC, and NLR to discriminate CMA group (n = 45) versus FGIDs and Control groups (n = 90).

**Table 4:** Comparison between Validity of CoMiSS, sEDN and hematological parameters to discriminate cow’s milk allergy group (n = 45) versus functional gastrointestinal disorders and Control groups (n = 90)

	AUC	*P*	95% CI	Cut off	Sensitivity	Specificity	PPV	NPV
**CoMiSS**	0.964	<0.001^1^	0.939–0.990	> 9	97.78	78.89	69.8	98.6
**sEDN level**	0.754	< 0.001^1^	0.657–0.834	> 14	86.67	60.00	52.0	90.0
**Total leukocyte count**	0.621	0.022^1^	0.515–0.728	> 8.8	66.67	53.33	41.7	76.2
**Mean platelet volume**	0.613	0.033^1^	0.499–0.727	> 8.9	62.22	53.33	40.0	73.8
**Absolute lymphocyte count**	0.661	0.002^1^	0.554–0.768	> 4000	60.0	53.33	39.1	72.7
**Absolute neutrophil count**	0.909	< 0.001^1^	0.855–0.962	> 3900	80.0	78.89	65.5	88.7
**Neutrophil lymphocyte ratio**	0.695	< 0.001^1^	0.599–0.791	>1	71.11	58.89	46.4	80.3

*AUC* Area Under a Curve

*p*-value Probability value

*CI* Confidence Intervals, *CoMiSS* Cow milk related symptom score

*sEDN* Serum Eosinophil Derived Neurotoxin, *NPV* Negative predictive value,

*PPV* Positive predictive value

^1^Statistically significant at *p* ≤ 0.05

### Correlation of sEDN with CoMiSS and hematological parameters

**Fig. 5:**
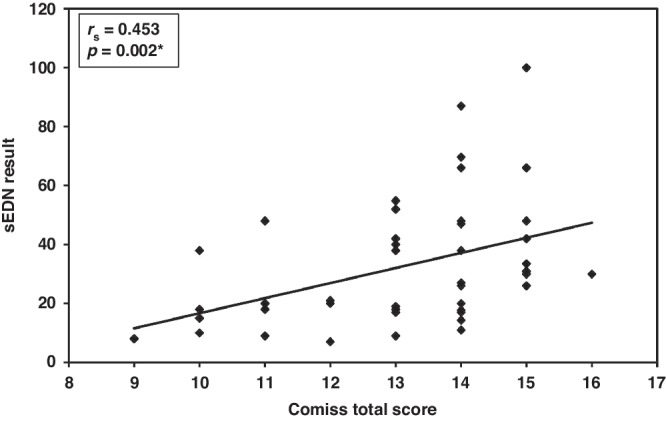
Correlation between Serum Eosinophil-Derived Neurotoxin and CoMiSS. A significant positive spearman’s coefficient correlation was observed between sEDN and CoMiSS

In the CMA group, a significant positive spearman’s coefficient correlation was found between sEDN and CoMiSS (**r**_**s**_ = 0.453 and *p* = 0.002) (Fig. 5). Among studied hematological parameters, only ANC showed a significant positive spearman’s coefficient correlation (**r**_**s**_ = 0.354 and *p* = 0.017). (Table 5)

**Table 5:** Correlation between the sEDN result with CoMiSS total score and hematological parameters in cow’s milk allergy and functional gastrointestinal disorders groups.

	sEDN result			
	Cow’s milk allergy		Functional GI disorders	
	r_s_	*p*	r_s_	*p*
**CoMiSS total score**	0.453	0.002^1^	0.191	0.208
**Total leukocyte count**	0.090	0.556	0.006	0.970
**Mean platelet volume**	0.014	0.926	-0.264	0.080
**Absolute lymphocyte count**	0.080	0.601	0.217	0.152
**Absolute neutrophil count**	0.354	0.017^1^	-0.049	0.747
**Neutrophil lymphocyte ratio**	0.0	0.999	-0.183	0.228

*sEDN* Serum Eosinophil-Derived Neurotoxin, *GI* Gastrointestinal, *r*_*s*_ Spearman coefficient.

*p*-value Probability value.

^1^Statistically significant at *p* ≤ 0.05.

### Linear regression analysis of independent variables for sEDN level

The univariate linear regression analysis of sEDN level with CoMiSS, CM specific IgE, and hematological parameters revealed that sEDN exhibited a statistical significance with CoMiSS and ANC (*p* = 0.004 and 0.015 respectively). On the other hand, no significance was observed between sEDN and CM specific IgE, ALC, AEC, MPV or NLR. For further evaluation of relationship between sEDN, CoMiSS and ANC, multivariate regression analysis was done which revealed CoMiSS as the only significant predictor for sEDN (*p* = 0.048) with no significance between sEDN and ANC (*p* = 0.199), (Table 6).

**Table 6:** Univariate and multivariate linear regression analysis of independent variables for sEDN level in cow’s milk allergy group (*n* = 45).

	Univariate linear regression		Multivariate	
	*p*	B (LL – UL 95%C. I)	*p*	B (LL – UL 95%C. I)
**CoMiSS total score**	0.004^1^	5.114(1.714–8.513)	0.048^1^	3.896(0.033–7.760)
**Cow milk specific IgE**	0.361	6.289(−7.46 to 20.03)		
**Mean platelet volume**	0.720	0.497(−2.28 to 3.27)		
**Absolute Eosinophilic count**	0.651	0.001(−0.004 to 0.002)		
**Absolute lymphocyte count**	0.688	0.001(−0.004 to 0.006)		
**Absolute neutrophil count**	0.015^1^	0.005(0.001 to 0.010)	0.199	0.003(−0.002 to 0.008)
**Neutrophil lymphocyte ratio**	0.059	11.409(−0.463 to 23.281)		

*sEDN* Serum Eosinophil Derived Neurotoxin, *CoMiSS* Cow milk related symptom, *score B* Unstandardized Coefficients, *C.I* Confidence interval, *LL* Lower limit, *UL* Upper Limit.

^1^Statistically significant at *p* ≤ 0.05.

## Discussion

Even though respiratory symptoms (wheezing, chronic cough), GI symptoms (regurgitation, vomiting, diarrhea, constipation, and bloody diarrhea), and general symptoms (poor growth and infantile colic) typically occur in 15-20% of infants, they can also be related to CMA diagnosis.^2^

CMA may be IgE-mediated, non-IgE-mediated, or mixed in which both IgE-mediated and cell-mediated immunological mechanisms are involved. Despite their frequency, non-IgE and mixed CMA are less understood.^18^ In our study, CM specific IgE was positive in only 33.3% of CMA group with no reported immediate clinical reactions. In contrast to IgE-mediated CMA, most cases of non-IgE and mixed CMA are diagnosed clinically, which is not always easy and often leads to a misdiagnosis or a diagnostic delay. This is owing to their potentially chronic presentation, delayed onset of symptoms, less obvious allergen link, and possibility for clinical overlap with GERD, infections, or FGIDs which are much more common in the first year of life with a reported prevalence of 25% to 77%.^7,19^ In addition, apart from an OFC with CM, there are currently no optimal diagnostic tests and biomarkers for non-IgE-mediated CMA.^20^ However, parents frequently refuse or delay OFC due to fear of a severe reaction.^21^

Failure to thrive (FTT) is an alarm symptom that can be caused by a variety of factors or underlying disease and requires a comprehensive diagnostic work-up and referral for full evaluation.^10^ In the current study, weight and weight for length were significantly lower in CMA group than the other 2 groups with prevalence of faltering growth in around one third of CMA infants. Despite CMA may have an impact on growth and lead to faltering growth, data are still limited.^8^

Considering that FGIDs infants and their families have a worse quality of life (QoL) and seek medical attention more frequently than asymptomatic controls, FGIDs have per definition no identifiable underlying organic cause.^22^ So, infants with poor weight gain or abnormal physical examination shouldn’t be diagnosed with FGIDs.^19^ This can explain the absence of significant difference between FGIDs and control groups in all anthropometric measurements in this study.

Patients with CMA often experience a wide range of symptoms that spread over many organ systems. FGIDs, on the other hand, only affect the GI tract and have general symptoms such as excessive crying and irritability. Thus, the presence of GI and/or general symptoms along with skin and/or respiratory signs increases the likelihood of CMA.^10^ Nevertheless, there is ongoing controversy regarding the relationship between CMA and FGIDs in the absence of further symptoms or signs of atopy.^19,23^ Therefore, it is crucial to search for a reliable test or biomarker for CMA diagnosis to prevent both overdiagnosis and underdiagnosis.

In our study, CoMiSS demonstrated higher statistical significance in the CMA group than FGIDs and control groups (with a median of 14). Given that the majority of our cases presented with multiple symptoms and that 46.66% of them had more than one system affection, this high score in the CMA group makes sense. Also, the median CoMiSS in healthy controls was comparable with reported results of CoMiSS cut-off score in healthy infants aged ≤ 6 months, which varies between 3 and 4,^24,25^ and CoMiSS in infants aged 6–12 months with an overall median of 3.^26^ The total CoMiSS was significantly higher in the FGIDs group (median 9) than the control group (median 4). However, no prior research has reported a cut-off score for CoMiSS in FGIDs.

Despite high median CoMiSS in CMA group in our study, ROC curve analysis of CoMiSS identified score > 9 as the best cut-off for differentiating between infants with CMA and without CMA (FGIDs and control groups) with 97.78% sensitivity and 78.89% specificity and AUC: 0.964 which was consistent with the most recent CoMiSS update with a cut-off score of ≥10 suggestive of CMA.^10^ According to eleven studies, a score of at least 12 indicates a favorable response to CMFD and the estimated sensitivity and specificity for CMA diagnosis were 20% to 77% and 54% to 92%, respectively.^2^ However, there is a disparity in the cut-off values of CoMiSS in different studies, ranging from ≥5.5 to ≥12, which can be attributed to differences in study design, some studies used symptoms as an inclusion criterion and calculated CoMiSS as supplementary information,^2,27,28^ while other studies used a CoMiSS above a specific cut-off as an inclusion criterion.^29,30^ This range of values indicates that the type of symptoms reported and the study design may have an impact on how CoMiSS operates. So, CoMiSS cannot be considered as a stand-alone CMA diagnostic tool.

Considering the lack of markers unique to CMA, CBC values may be regarded as a useful tool for inflammatory diagnosis and monitoring in numerous chronic disorders as well as in allergic reactions, where MPV and NLR have been investigated as inflammatory markers with contradictory findings.^11,31–33^

In the current study, MPV and TLC, though with low sensitivity and specificity, revealed a statistically significant difference between the CMA and control groups but not between the CMA and FGIDs groups. Similarly, Çam^34^ found that the MPV values of the allergic proctocolitis (AP) group were statistically higher than the control group. In contrast, Dogan and Sevinc^35^ found no significant difference in MPV levels of infants with and without CMA. These findings suggest that alterations of MPV levels may be easily affected by the type and severity of inflammation.

Few studies have assessed NLR in children with most of these studies focusing on NLR in asthma.^36,37^ Like studies assessing MPV levels, varied results for NLR in asthma have been documented.^38,39^ Furthermore, there was no significant difference in NLR between infants with CMA and controls according to Çam.^34^ In our results, despite NLR in the CMA group being statistically significant compared to the other groups, ROC curve showed low sensitivity and specificity. Therefore, its role in CMA diagnosis is debatable.

About 60% of all leukocytes in the bloodstream are neutrophils, which play a significant role in type 1 and type 3 immunological responses but have a controversial role in type 2 immunity.^40,41^ Severe allergies, autoimmune and autoinflammatory diseases are influenced by neutrophil dysregulation.^42^ Furthermore, in FPIES, positive responses to the OFC are usually followed by a rise in the ANC of more than 3500 cells/mm3.^43^ Our findings were in the same line as ANC showed a higher statistically significant difference in CMA group than other groups, and ROC curve revealed 80% sensitivity and 78.89% specificity at a cut-off point >3900 cells/mm3. Likewise, Kimura et al.^44^ reported a significant increase in ANC in OFC-positive than in OFC-negative subjects. On the other hand, any bacterial infection, inflammation, burns, cancer, and certain medications, like cortisol, also cause an increase in neutrophils.^45^ Furthermore, blood samples that are either EDTA-based or insufficiently anticoagulated with heparin or citrate might result in platelet clumping in an automated cell counter leading to falsely elevated neutrophil count (factitious neutrophilia).^46^ As a result, ANC monitoring for infants with CMA is suggested. However, its usefulness as a CMA biomarker is questionable.

Non-IgE FA is characterized by increased intestinal permeability and inflammation, which lead to granulocytes and eosinophils migrating to the intestinal lumen. Fecal markers are therefore a helpful diagnostic tool for inflammatory disorders in gastroenterology and a non-invasive means of assessing intestinal inflammatory responses. Additionally, the identification of fecal biomarkers is becoming increasingly interesting due to the absence of reliable diagnostic techniques.^17^

Numerous studies have investigated the use of different fecal biomarkers for CMA diagnosis, including fecal IgA, EDN, eosinophilic cationic protein (ECP), and fecal calprotectin (FC).^17^ Unfortunately, studies regarding fecal EDN in non-IgE-mediated CMA children are controversial and intestinal permeability appears to interact with the composition of the microbiota, which has been implicated in the development of food allergies.^47^ fEDN level was higher in CMA patients compared to controls.^1,17^ Although this finding seems promising, these differences were not statistically significant.

The most reliable marker of allergic inflammation is eosinophils, which have big cytoplasmic granules that contain proteins such major basic protein, eosinophil protein X, EDN, and ECP. Numerous inflammatory and allergic conditions, including atopic dermatitis, asthma, and other conditions can raise the number of eosinophils in the blood.^48,49^ Additionally, elevated EDN and ECP levels have been reported in asthma patients, and these levels are associated with exacerbating asthma symptoms. Therefore, EDN and ECP may aid in the diagnosis and monitoring of asthma.^50^

In the same line, Çam^34^ found significantly higher sECP levels in infants with CMA compared to controls (sensitivity 60.7% and specificity 97.5%). Furthermore, comparable results were observed by some studies.^51–53^ Nonetheless, several studies have found that sEDN is a more accurate indicator of disease severity than sECP.^14,50^ In a prior study, sEDN levels were assessed in 4 allergic diseases (bronchial asthma, atopic dermatitis, allergic rhinitis, and FA) in children aged 6 to 12 years, and the ROC curve of sEDN revealed 81.2% sensitivity, 69.8% specificity, and AUC: 0.790.^14^

But as far as we know, there are no studies in the literature on the role of sEDN in the diagnosis of infants with CMA or the relationship between sEDN, CoMiSS and hematological parameters. In our study, sEDN at a cut-off point > 14 ng/mL demonstrated 86.67% sensitivity, 60.00% specificity and AUC: 0.754 in differentiating infants with CMA than those without CMA. Additionally, Spearman’s coefficient revealed a significant positive correlation between sEDN with both CoMiSS and ANC. However, multivariate linear regression analysis found that CoMiSS was the only parameter that statistically and independently influenced the sEDN level in the CMA group. CoMiSS as an acknowledged awareness tool, demonstrated good screening performance in our study; however, its high sensitivity was attributed to the fact that most cases had multiple symptoms, increasing its total score. Furthermore, the heterogeneous presentation of CMA and the subjectivity of parental perception of severity and duration of crying (which may be over-reported) may limit the diagnostic value of CoMiSS in CMA diagnosis.

Therefore, given the increasing prevalence of CMA, we recommend more research on the role of sEDN in CMA diagnosis including participants with a broad range of symptoms with evaluation of its level prior to and following CMFD, and to evaluate sEDN role in disorders other than FGIDs which may overlap with CMA diagnosis. Additionally, to fully understand their significance in CMA diagnosis, it is advisable to correlate sEDN with CoMiSS and hematological parameters, particularly ANC.

The main strengths of our study were that we are the first study to investigate sEDN in CMA infants. Also, we compared sEDN in CMA infants with infants diagnosed with FGIDs which are much more common in the first year of life and have similar manifestations to CMA, in addition to healthy controls. Moreover, all samples were withdrawn in absence of any acute illness, dietary interventions, or drug intake to avoid their possible impact on results. Finally, we correlate sEDN with both basic hematological parameters and CoMiSS which is the available clinical awareness tool for CMA.

## Limitations of the study

There were some limitations in this study. As there have been no studies evaluating sEDN in infants with CMA, we could not compare our results with others. Also, relatively few studies evaluated hematological parameters in CMA, so we compared our findings to only a small number of studies.

## Conclusions

sEDN had a high diagnostic performance in infants with CMA. Therefore, sEDN is recommended as a potential biomarker for CMA diagnosis. Also, CoMiSS demonstrated a strong ability to distinguish CMA from both healthy and FGIDs-affected infants at a cut-off point >9, highlighting its important role as an awareness tool for CMA. Furthermore, CoMiSS was the only significant predictor related to sEDN level in infants with CMA. Additionally, ANC demonstrated the best performance among the hematological parameters; therefore, more research is necessary to investigate ANC’s potential as a simple, inexpensive, and widely available biomarker.

## Data Availability

All datasets presented in this study are included in the article.
